# Veterinary Register

**Published:** 1902-05

**Authors:** 


### VETERINARY REGISTER.

[See Editorial, Journal of Comparative Medicine and Veterinary Archives, July, 1901, page 438.]

[In the following list the names and data printed without comment are those furnished to us by postal card with the signature of the individual.

Those with an asterisk [*] are those to whom we have sent, by United States mail, an envelope with prepaid postage and a request for return if not delivered in ten days; as the envelopes have not been returned, we presume that the letter has been delivered, that the individual in question exists, but that he has neglected to return the enclosed postal card with the information requested.—Ed. Journal.]

### PENNSYLVANIA.

(Continued from page 268.)

| *Name.* | *Street,* | *Town.* | *College, Graduated.* | *County, Registered.* |
|---|---|---|---|---|
| Stephens, J. | ...... | Nicholson | * | ...... |
| Sterling, Cyrus C. | ...... | Summer Hill Twp. | * | ...... |
| Sterner, C. D. | ...... | Brodbecks | * | ...... |
| Stevens, A. E. | ...... | Keeneyville | * | ...... |
| Stevens, Elwood | 2d st Pike near Borbeck | Fox Chase | * | ...... |
| Stevens, N. O. | ...... | Girard | * | ...... |
| Stevenson, J. F. | ...... | Greenville | * | ...... |
| Stewart, Archib'd G. | ...... | Conoquenessing Twp. | * | ...... |
| Stickles, Samuel | ...... | English Centre | * | ...... |
| Stockdill, P. F. | ...... | Rural Valley | * | ...... |
| Stoever, J. F. | ...... | Fredericksb'g | * | ...... |
| Storm, Wm. J. | 1825 S. 18th st | Philadelphia | * | ...... |
| Stouffer, David C. | ...... | Hopewell | * | ...... |
| Stroughton, O. P. | ... .. | Scott Twp. | * | ...... |

| *Name.* | *Street.* | *Town.* | *College, Graduated.* | *County, Registered.* |
|---|---|---|---|---|
| Stover, John B. | ...... | Shady Grove | Ontario Vet., 1893 | Franklin, 1893 |
| Stover, John F. | ...... | Fredericksb'g | * | ...... |
| Stover, Mahlon G. | ...... | Nazareth | * | ...... |
| Strang, Frank | ...... | Westfield | * | ...... |
| Strange, Wm. | ...... | Pittston | * | ...... |
| Strayer, Henry | ...... | Springfield Twp. | * | ...... |
| Strayer, Wm. | 655 S. Duke st | Lancaster | * | ...... |
| Strickler, Chas. M. | ...... | Greencastle | * | ...... |
| Strouse, Joseph | ...... | Keller's Church | * | ...... |
| Sturge, E. | 420 Spruce st | Scranton | * | ...... |
| Sullivan, Dennis O. | ...... | Devon | ...... | Chester, 1889 |
| Summers, George L. | ...... | Duffield | * | ...... |
| Summy, H. B. | ...... | Manheim | * | ...... |
| Super, Daniel H. | ...... | Warrensville | Ontario Vet., 1895 | Lycoming, 1899 |
| Sutton, Bruce S. | 130 Edwards ct | Scranton | ...... | Lackawanna, 1889 |
| Swank, George K. | Centre st | E. Mauch C'k | V. D. U. Pa., 1894 | Carbon, 1894 |
| Swartz, Henry | ...... | New Oxford | * | ...... |
| Swartz, Jacob N. | ...... | Redland | * | ...... |
| Swartz, Levi B. | ...... | Wismer | * | ...... |
| Sweeley, D. W. | ...... | Jersey Shore | * | ...... |
| Sweeley, Elmer E. | ...... | Jersey Shore | * | ...... |
| Sweet, John B. | ...... | Buell | * | ...... |
| Swift, S. J. | Chestnut st | Franklin | National Vet., 1895 | Venango, 1895 |
| Swords, Leonard | ...... | W. Brownsville | * | ...... |
| Sypher, Henry J. | ...... | Milton | * | ...... |
| Tallman, B. H. | 129 W. Church | Williamsport | V. D. U. Pa., 1900 | Lycoming, 1900 |
| Taylor, F. | ...... | Sewickley | * | ...... |
| Taylor, Wm. | 22d & Cambria | Philadelphia | * | ...... |
| Tebay, Milton | ...... | Centre Twp. | * | ...... |
| Temple, S. W. | ...... | Warsaw | * | ...... |
| Terry, Edward E. | Welsh road | Holmesburg | V. D. U. Pa., 1893 | Bucks, 1893 |
| Terry, Harry C. | Bellone av | Langhorne | American Vet., 1894 | Bucks, 1894 |
| Thomas, John | ...... | Greensburg | * | ...... |
| Thomas, Thomas | ...... | Troxelville | * | ...... |
| Thompson, Wm. C. | ...... | Berwyn | * | ...... |
| Thorpe, J. W. | ...... | Sugar Grove | * | ...... |
| Thudicum, C. L. | ...... | Wayne | * | ...... |
| Tilyon, E. D. | ...... | Russell | * | ...... |
| Timberman, J. H. | 86 Union st | Wilkesbarre | * | ...... |
| Tintsman, John Z. | 335 E. Girard av | Philadelphia | V. D. U. Pa., 1888 | Philadelphia, 1889 |
| Tippery, Samuel | ...... | Foxburg | * | ...... |
| Tomlinson, W. J. | 23 State st | Williamsport | * | ...... |
| Totten, J. M. | ...... | Mechanicsburg | * | ...... |
| Tower, E. E. | ...... | Montrose | National Vet., 1894 | Susquehanna,1894 |
| Trego, Daniel | ...... | Rockville | * | ...... |
| Treichler, Albert J. | ...... | Collegeville | * | ...... |
| Tressler, Henry J. | ...... | Bellefonte | * | ...... |
| Trexler, J. P. | ...... | Shamokin Dam | * | ...... |
| Trone, Hezekiah | ...... | Hanover | * | ...... |
| Troutman, Elias | ...... | Rehrersburg | * | ...... |
| Tully, Edgar M. | 1945 Franklin | Philadelphia | * | ...... |
| Turnbull, W. A. | 815 Ligonier av | Latrobe | * | ...... |
| Turner, Henry W. | ...... | Lahaska | * | ...... |
| Uibel, C. B. | ...... | Bowmansville | * | ...... |
| Ulsh, H. M. | ...... | McClure | * | ...... |
| Ulsh, H. S. | ...... | Lewistown | * | ...... |
| Underhill, Benj. M. | 41 E. Front st | Media | V. D. U. Pa., 1895 | Philadelphia, 1895 |
| Urberger, Simon | ...... | Swatara Twp. | * | ...... |
| Van Cise, George | ...... | Sharon Center | * | ...... |
| Vandegrift, John F. | State st | Newtown | V. D. U. Pa., 1888 | Bucks, 1889 |
| Vanderbilt, Peter | 525 W. 3d st | Williamsport | * | ...... |
| Van Dyke, E. G. | ...... | E. Canton | * | ...... |
| Van Horn, G. L. | ...... | North Point | * | ...... |
| Van Horn, J. W. | ...... | Montoursville | * | ...... |
| Varnas, Joseph | ...... | E. Salem | * | ...... |
| Varnum, Enoch | ...... | North Hope | * | ...... |

| *Name.* | *Street.* | *Town.* | *College, Graduated.* | *County, Registered.* |
|---|---|---|---|---|
| Vasey, J Price | 1927 N. Camac | Philadelphia | ...... | Philadelphia, 1889 |
| Vetter, John J. | Cherry av near Liberty av | Pittsburg | * | ...... |
| Vetter, Stephen A. | ...... | Pittsburg | * | ...... |
| Vogel, Peter | ...... | Berlin | * | ...... |
| Von Lang, Otto | 39th & Hav'f'd | Philadelphia | * | ...... |
| Waidner, C. J. | Main st | Hellertown | N. Y. C. V. S., 1889 | North'pton, 1889 |
| Waldron, R. M. | 111 Talbott av | Greensburg | Ontario Vet., 1887 | Westmorel'd, 1901 |
| Waldron, Thos. M. | 9 E. Peters st | Uniontown | * | ...... |
| Wall, Miles | ...... | Curwensville | * | ...... |
| Wallace, S. J. | 605 Water st | Warren | * | ...... |
| Wallace, T. C. | 205 E. 2d st | Warren | * | ...... |
| Walter, Charles R. | ...... | Conyngham | * | ...... |
| Wareham, Henry | ...... | Carlisle | * | ...... |
| Warmcastle, Chas. A. | ...... | Pittsburg | * | ...... |
| Warren, Wm. H. | ...... | Quarryville | * | ...... |
| Washburn, Silas S. | ...... | Smethport | * | ...... |
| Wasser, Benj. H. | ...... | Applebachsville | * | ...... |
| Wasser, Charles | ...... | Durham | * | ...... |
| Watkins, Frank | ...... | Philadelphia | * | ...... |
| Waugh, David | 1 Robinson st cor 5th av | Pittsburg | Indiana Vet., 1897 | Washington, 1889 |
| Waugh, James A. | 1 Robinson st cor 5th av | Pittsburg | Ontario Vet., 1882 | Allegheny, 1890 |
| Waugh, Wm. J. | 111 W.Wheel'g | Washington | Ontario Vet., 1882 | Washington, 1900 |
| Weand, E. W. | Main av & Lee st | Macungie | * | ...... |
| Weamer, S. C. | ...... | Marion Centre | * | ...... |
| Weaver, George | ..... | Lone Pine | * | ...... |
| Weaver, Samuel W. | ...... | East Earl | * | ...... |
| Weaver, V. B. | Broadway | Bangor | ...... | Lehigh, 1878 |
| Weber, S. E | ...... | Lancaster | Ontario Vet., 1884 | Lancaster |
| Webster, Richard G. | 7th & Kerlin sts | Chester | V. D. U. Pa., 1887 | Delaware |
| Wehr, G. A. | ...... | Denver | * | ...... |
| Weicksel, H. J. S. | 300 N. Rock st | Shamokin | V. D. U. Pa., 1894 | Northumb'd, 1894 |
| Weidenhammer, A. G. | ...... | Kutztown | * | ...... |
| Weiger, John | ...... | Allen Twp. | * | ...... |
| Weikel, Lemuel | ...... | New Columbia | * | ...... |
| Weinberg, H. H. | 1202 N. Fr'klin | Philadelphia | * | ...... |
| Weir, James B. | 121 Market st | Kittanning | Gr'd Rapids Vet., 1900 | Indiana, 1891 |
| Weir, James B. | ...... | Smicksburg | * | ...... |
| Weir, Samuel D. | ...... | Fleetwood | * | ...... |
| Well, J. B. | ...... | Terre Hill | * | ...... |
| Weller, J. O. | ...... | Meyersdale | * | ...... |
| Welliver, E. F. | ...... | Shamokin | * | ...... |
| Welliver, G. H. | ...... | Bloomsburg | Ontario Vet., 1893 | Columbia, 1894 |
| Wenger, Z. B. | ...... | Akron | * | ...... |
| Wentz, W. T. S. | ...... | Philadelphia | * | ... .. |
| Werntz, W. B. | 4531 Lan'ter av | Philadelphia | * | ...... |
| Wetzel, F. F. | ... .. | Millheim | * | ...... |
| Wheeler, J. J. | ...... | Lander | * | ...... |
| Whitaker, C. J. | ...... | Girard | * | ...... |
| Whitcomb, J. | ...... | Centre Square | * | ...... |
| White, David J. | ...... | N. P. I., Wash. Co. | * | ...... |
| White, James S. | ...... | Millerstown | * | ...... |
| White, Thomas | 1210 S. 28th st | Philadelphia | V. D. U. Pa., 1900 | ...... |
| Whiteside, John D. | Main ab. State | Parkesburg | * | ...... |
| Whiting, F. L. | ...... | Harmonsburg | * | ...... |
| Whiting, I. N. | ...... | Mansfield | * | ...... |
| Whitling, W. H. | ...... | Knox | * | ...... |
| Widener, E. W. | ...... | Pittston | * | ...... |
| Widmer, John | S. Main st | Carbondale | ...... | Lackawanna, Monroe, 1889 |
| Widner, Edward W. | ...... | Pittston | * | ...... |
| Widner, E. W. | .. ... | Stroudsburg | * | ...... |
| Wieand, V. H. | 118 N. 6th st | Allentown | * | ...... |
| Wier, A. W. | Clinton st | Greenville | Ohio Vet. Med., 1895 | Mercer |

